# Targeting Mutant p53 by a SIRT1 Activator YK-3-237 Inhibits the Proliferation of Triple-Negative Breast Cancer Cells

**DOI:** 10.18632/oncotarget.1070

**Published:** 2013-07-05

**Authors:** Yong Weon Yi, Hyo Jin Kang, Hee Jeong Kim, Yali Kong, Milton L. Brown, Insoo Bae

**Affiliations:** ^1^ Department of Oncology, Lombardi Comprehensive Cancer Center, Georgetown University, Washington, DC; ^2^ Department of Radiation Medicine, Lombardi Comprehensive Cancer Center, Georgetown University, Washington, DC; ^3^ Center for Drug Discovery, Georgetown University, Washington, DC

**Keywords:** mutant p53 (mtp53), deacetylation, SIRT1, activator, triple-negative breast cancer (TNBC)

## Abstract

Many types of mutations in tumor suppressor p53 are oncogenic through gain-of-function. Therefore, targeting mutant p53 (mtp53) is a promising therapeutic approach to fight against many types of cancers. We report here a small molecule compound YK-3-237 that reduces acetylation of mtp53 and exhibits anti-proliferative effects toward triple-negative breast cancer (TNBC) cells carrying mtp53. YK-3-237 activates SIRT1 enzyme activities *in vitro* and deacetylation of both mtp53 and wild type p53 (WTp53) in a SIRT1-dependent manner. Deacetylation of mtp53 resulted in depletion of mtp53 protein level and up-regulated the expression of WTp53-target genes, PUMA and NOXA. YK-3-237 also induces PARP-dependent apoptotic cell death and arrests the cell cycle at G2/M phase in mtp53 TNBC cells. Taken together, our data suggest that targeting acetylation of mtp53 is a potential target to treat human cancers.

## INTRODUCTION

TNBCs are immunohistologically characterized by lack of estrogen receptor (ER) and progesterone receptor (PR) as well as human epidermal growth factor receptor 2 (HER2) amplification, and ~20% of all breast cancers are identified as TNBCs [1]. TNBC tumors are a group of heterogeneous cancers and substantial overlapped with basal-like breast cancer [1]. Although early TNBCs have higher response rates to neoadjuvant chemotherapy, patients with advanced disease show poor response and rapid progression of disease than patients with other types of breast cancers [2]. Currently, there are no therapies that target TNBCs [2,3].

Breast tumors with p53 mutations were mostly classified into basal-like or HER2-amplified subgroup, while luminal subgroup of breast cancers almost exclusively expresses WTp53 [4]. Tumor suppressor protein p53 is a cellular guardian and mutations of p53 are found ~50% of human cancers [5-8]. In fact, p53 is originally described as a tumor protein (or oncoprotein) and subsequent investigations have revealed that WTp53 has roles as a tumor suppressor and protects cells from malignant transformation [5-8]. In general, mutation of p53 results in stabilization and accumulation of mtp53 proteins in cells, while WTp53 is tightly regulated by post-translational modification coupled with proteasomal degradation. First reports appeared to support that mutation of p53 simply causes a loss of tumor suppressor function. Recent advances, however, have demonstrated that mtp53 possess pro-oncogenic potential through gain of functions such as transcriptional repression and activation, coaggregation with other tumor suppressors, and so on [5-8]. Therefore, targeting mtp53 pathways may represent a promising approach in the development of novel anti-cancer agents [4,6]. Because mtp53 has many types of mutations and functions, distinct strategies will be required for therapeutic targeting [6].

Silent information regulator 2 (Sir2) proteins, or surtuins (SIRTs), are evolutionally conserved enzyme family acting as protein deacetylases/ADP ribosyltransferases [9-12]. At least 7 enzymes of this class have been identified in humans (SIRT1-7). Among these proteins, class I sirtuins (SIRT1-3) and SIRT5 showed deacetylase activity [9-12]. It has been reported that SIRT1, SIRT2, SIRT3, SIRT4 and SIRT6 appear to act as tumor suppressors in certain conditions [13-17]. Since varied reports on tumor suppressor function of SIRT1 have been reported [18,19], SIRT1 activators could be useful for treatment or prevention of certain type of cancers [9-12]. In addition to resveratrol, a series of compounds are reported as potent SIRT1 activators [20-24]. More recently, years-long debates on the activation of SIRT1 by these compounds [25-28] were resolved by two independent groups [29,30].

YK-3-237 is originated as a boronic acid chalcone analog of combretastatin A-4 (CA-4) [31]. Unlike CA-4, YK-3-237 did not bind to tubulin *in vitro* [31] and exhibited potent anti-proliferative activity toward a broad range of NCI cancer cell lines with unknown mechanism [31]. Here we report that YK-3-237 deacetylates mtp53 through SIRT1 and inhibits the proliferation of TNBC cell lines carrying mtp53.

## RESULTS

### YK-3-237 inhibits the proliferation of TNBC cells

**Figure 1:**
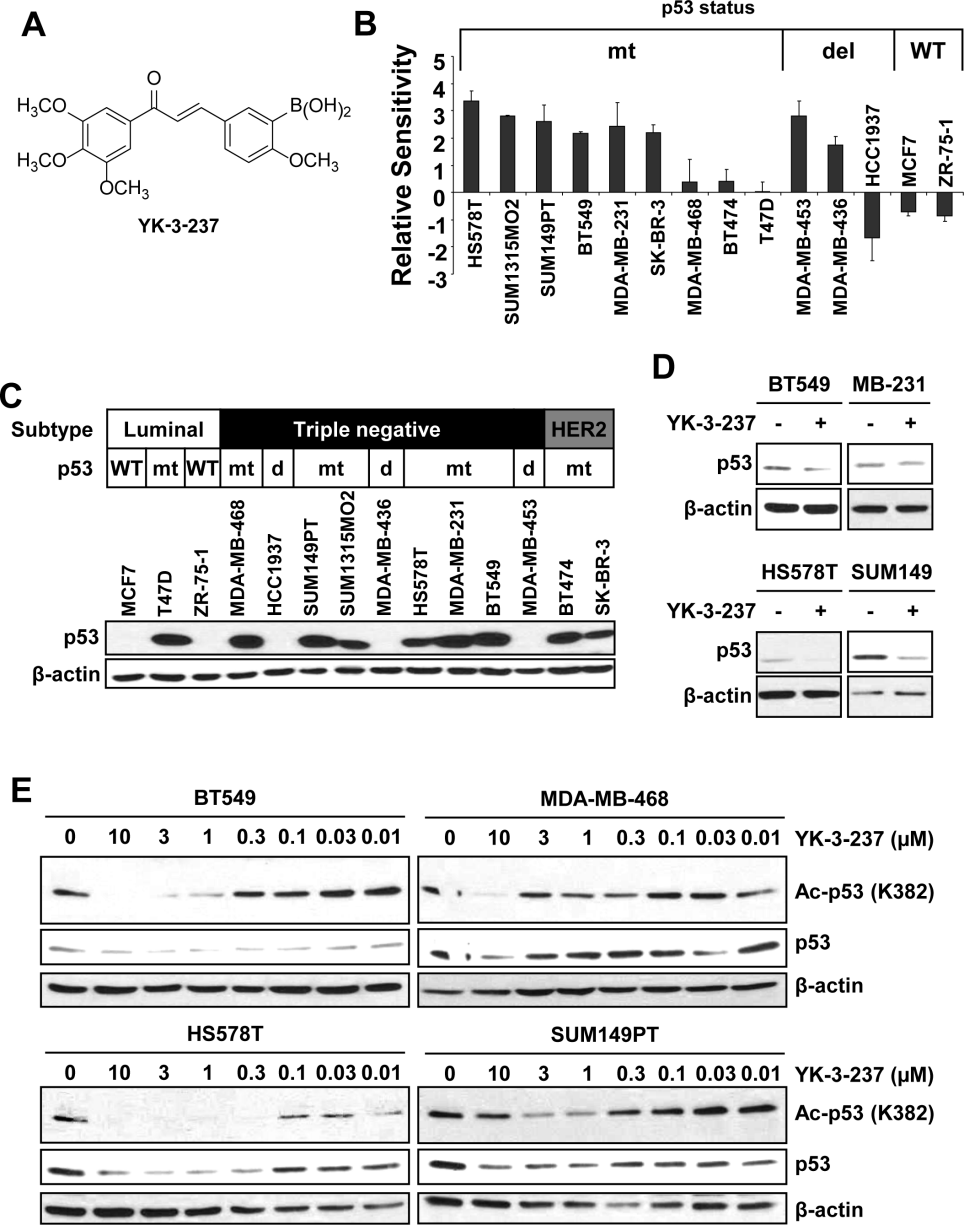
YK-3-237 reduces the proliferation and acetylation of mtp53 in breast cancer cell lines A, structure of YK-3-237. B, relative sensitivities of breast cancer cell lines to YK-3-237. Breast cancer cells were treated with increasing amount of YK-3-237 for ~72 hr and the viable cells were measured by MTT assay. Relative sensitivities were calculated as described in Materials and Methods. The EC_50_ value of each cell line was plotted in Sup Figure 1. Data are presented as mean ± SEM from two independent experiments performed in triplicate. C, western blot analysis of breast cancer cell lines. Cell lysates from each cell line were analysed by indicated antibodies. D, effect of YK-3-237 on the level of mtp53. Cells were treated with 1 µM of YK-3-237 for 24 hr and western blot analysis was performed with indicated antibodies. E, YK-3-237 reduces acetyl-mtp53. Cells were treated with increasing concentrations of YK-3-237 for 24 hr and cell lysates were subjected to western blot analysis with indicated antibodies. C~E, β-actin was used as a loading control.

Previously it has been reported that YK-3-237 (Figure 1A) exhibited potent anti-proliferative activity toward a broad range of NCI cancer cell lines with unknown mechanism [31]. To further determine the anti-proliferative effects of YK-3-237, we performed the cell viability assay with a panel of breast cancer cell lines. Cells were treated with increasing concentration of YK-3-237 up to 72 hr and viable cells were measured by MTT assay. Notably, YK-3-237 exhibited the anti-proliferative activities toward most of the breast cancer cell lines tested at submicromolar concentration (Table 1 and Sup Figure 1). As shown in Figure 1B, YK-3-237 more preferentially inhibited the proliferation of breast cancer cell lines carrying mtp53. As previously reported [4], most of TNBC cell lines in this study are expressing mtp53 (Table 1). Western blot analysis showed that the levels of p53 protein (data not shown) are highly elevated in TNBC cell lines carrying mutations of p53 gene (Figure 1C). Although cells with WTp53 such as MCF7 and ZR-75-1 expressed detectable levels of p53 protein, the levels of mtp53 protein are much higher than that of WTp53. As expected, expression of ERα was not detected in TNBC cell lines (Sup Figure 2). Notably, no significant difference in the level of SIRT1 protein was observed in our breast cancer cell line panel (Sup Figure 2). To determine the effect of YK-3-237 on the level of mtp53, western blot analysis was further performed with cell lysates from TNBC cells treated with 1 µM of YK-3-237 for 24 hr. We found that YK-3-237 (1 µM) reduced the level of mtp53 protein in all TNBC cell lines tested after 24 hr treatment (Figure 1D).

**Table 1:** Summary of breast cancer cell panel and EC_50_ for YK-3-237

	Cell line	EC_50_ ± SEM (µM)	p53 Status [32]
TNBC	HS578T	0.160 ± 0.043	V157F
	MDA-MB-453	0.241 ± 0.086	Homozygous deletion (exon 10/11)
	SUM1315MO2	0.253 ± 0.028	C135F
	SUM149PT	0.289 ± 0.066	M237I
	BT549	0.353 ± 0.017	R249S
	MDA-MB-231	0.431 ± 0.136	R280K
	MDA-MB-436	0.501 ± 0.062	E204fsX45 (Del)
	MDA-MB-468	1.436 ± 0.754	R273H
	HCC1937	5.031 ± 2.010	R306X (Del)
Luminal	T47D	1.573 ± 0.370	L194F
	MCF7	2.402 ± 0.256	WT
	ZR-75-1	3.822 ± 0.967	WT
HER2	BT474	1.249 ± 0.372	E285K
	SK-BR-3	0.346 ± 0.066	R175H

### YK-3-237 deacetylates mtp53 in TNBC cell lines

Previously it has been reported that the stability of WTp53 is post-translationally regulated by acetylation at K382 residue [33]. Recently, mtp53 has also been reported to be regulated by acetylation [34]. Based on these findings, we further analysed the acetylation status of mtp53 in TNBC cell lines treated with YK-3-237 by western blot analysis. Twenty four hour treatment of YK-3-237 reduced both the acetylation of K382 and the level of mtp53 in a dose-dependent manner in mtp53 TNBC cell lines (Figure 1E). We observed that treatment of YK-3-237 had little or no significant effect on the level of SIRT1, one of the deacetylases for p53 [35, 36], in mtp53 TNBC cell lines upto 10 µM (Sup Figure 3A). The deacetylation of mtp53 was observed as early as 4 hr after treatment of YK-3-237 without significant reduction in mtp53 level (Sup Figure 3B).

**Figure 2:**
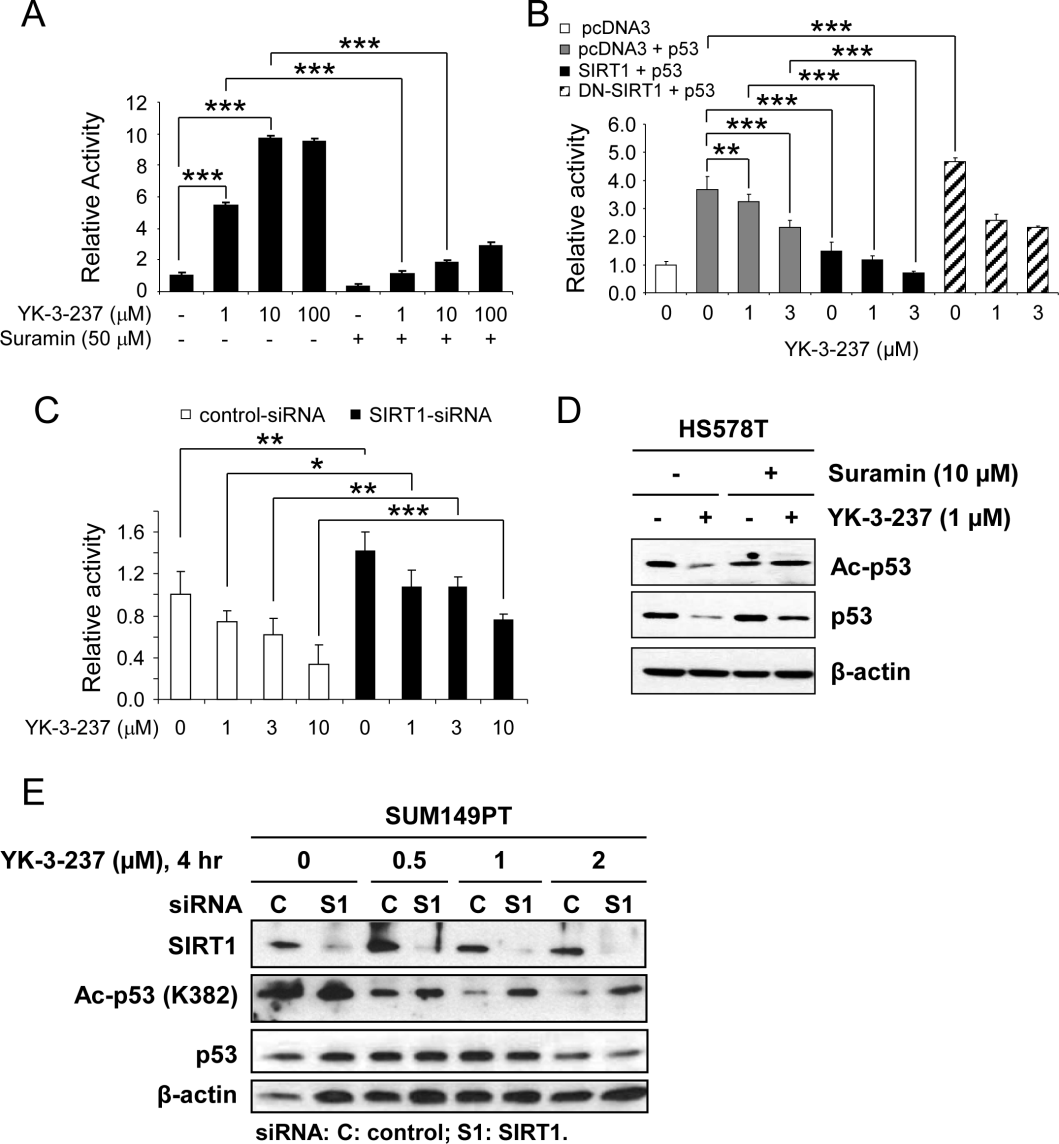
The effect of YK-3-237 on the activity of SIRT1 A, the effect of YK-3-237 on the enzyme activity of purified human SIRT1 enzyme *in vitro*. SIRT1 enzyme assay was performed as described in the Materials and Methods. Data are presented as mean ± SD. *** P < 0.001. B, the effect of YK-3-237 on the SIRT1-mediated repression of WTp53 transcriptional activity. MCF7 cells were transfected with indicated plasmid DNAs and further treated with YK-3-237 for 24 hr. Luciferase reporter activity was measured as described in the Materials and Methods. Data are presented as mean ± SD. **P < 0.01 and ***P < 0.001. C, SIRT1-KD reverses the YK-3-237-mediated repression of WTp53 transcriptional activity. MCF7 cells were transfected with siRNA and p53-Luc and further treated with YK-3-237 for 24 hr. Relative luciferase activity was determined as in B. Data are presented as mean ± SD.*P < 0.05, **P < 0.01, and *** P < 0.001. D, suramin reduces the YK-3-237-mediated deacetylation of mtp53. HS578T cells, pretreated with suramin for 1 hr, were further treated with YK-3-237 for 23 hr. Western blot analysis was performed as indicated. β-actin was used as a loading control. E, the effect of YK-3-237 on the SIRT1-mediated deacetylation of mtp53. SUM149PT cells were transfected with either control (C)- or SIRT1 (S1)-siRNA for 3 days, re-seeded and further treated with YK-3-237 for 4 hr. Western blot analysis was conducted with indicated antibodies. β-actin was used as a loading control.

Since SIRT1 is a well-known deacetylase for p53 on K382 residue, we further addressed whether YK-3-237 affects SIRT enzyme activity by *in vitro* SIRT assay with a fluorophore-conjugated peptide substrate. As shown in Figure 2A, YK-3-237 activated SIRT1 enzyme activities in a dose-dependent manner. Under this condition, a SIRT1/2 inhibitor suramin [37] antagonized YK-3-237-mediated SIRT1 activation. Interestingly YK-3-237 was more potent to activate SIRT1 activity than resveratrol and maximal activation was observed at 10 µM (Sup Figure 4A). Moreover, YK-3-237 effectively reduced the survival of SUM149PT cells as compared resveratrol in a long-term survival assay (Sup Figure 4B). YK-3-237 also activated the SIRT2 enzyme *in vitro* and enhanced the deacetylation of α-tubulin (K40) in HS578T cells (Sup Figure 4C and D).

To exclude potential artifacts from the *in vitro* assay, we further confirmed SIRT1 activation by YK-3-237 using a p53-Luc reporter gene assay (Figure 2B-C). Since acetylation of WTp53 has been known to induce its stabilization and transcriptional activation [33], we used WTp53 to monitor deacetylase activity of SIRT1 by YK-3-237. MCF7 cells were transfected with p53-Luc reporter plasmid and WTp53 expression vector in the presence or absence of SIRT1 or dominant negative (DN)-SIRT1 expression vector [38] and further treated with YK-3-237. As expected, YK-3-237 repressed the WTp53-mediated activation of p53-Luc reporter gene in a dose-dependent manner (Figure 2B, lanes 1~4). Under this condition, co-expression of SIRT1 reduced the p53-Luc reporter activity induced by WTp53 and co-treatment of YK-3-237 further enhanced the SIRT1-mediated repression of p53-Luc reporter (Figure 2B, lanes 5~7). On the contrary, DN-SIRT1 did not repress the p53-mediated reporter gene activation. In addition, repression of the p53-Luc reporter by YK-3-237 was limited in the presence of DN-SIRT1 (Figure 2B, lanes 9 and 10).

The effect of SIRT1 knockdown (SIRT1-KD) on the WTp53-mediated transcription was also tested in MCF7 cells. MCF7 cells were transfected by either control- or SIRT1-siRNA followed by transfection of p53-Luc and further treated with increasing amounts (1~10 µM) of YK-3-237. Under these conditions, SIRT1-KD slightly induced p53-Luc reporter activity (Figure 2C). While YK-3-237 reduced the p53-Luc reporter activity in control-siRNA-transfected cells, SIRT1-KD antagonized YK-3-237-mediated repression of p53-Luc activity. These results suggest that the repression of WTp53 transcriptional activity by YK-3-237 is, at least in part, dependent on the presence of functional SIRT1.

SIRT1-dependent deacetylation of mtp53 was further investigated in two mtp53 cell lines, HS578T and SUM149PT. First, HS578T cells were pre-treated with 10 µM of suramin for 1 hr and further treated with 1 µM of YK-3-237 for 23 hr. As shown in Figure 2D, pre-treatment of suramin reversed both deacetylation of mtp53 (K382) and reduction of mtp53 protein. Second, the effect of SIRT1-KD on the YK-3-237 activity was determined in SUM149PT cells. After transfection of either control- or SIRT1-siRNA, the cells were further treated with increasing amount of YK-3-237 for 4 hr. Under this condition, knockdown of SIRT1 increased acetyl-mtp53 in SUM149PT cells (Figure 2E). In addition, knockdown of SIRT1 reversed deacetylation of mtp53 by YK-3-237 (Figure 2E). Taken together, these results suggest that YK-3-237 activates the SIRT1 enzyme activity *in vitro* and deacetylates both wild type and mutant p53 in cells in a SIRT1-dependent manner.

### YK-3-237 induces WTp53-target gene expression in TNBC cell lines carrying mtp53

**Figure 3:**
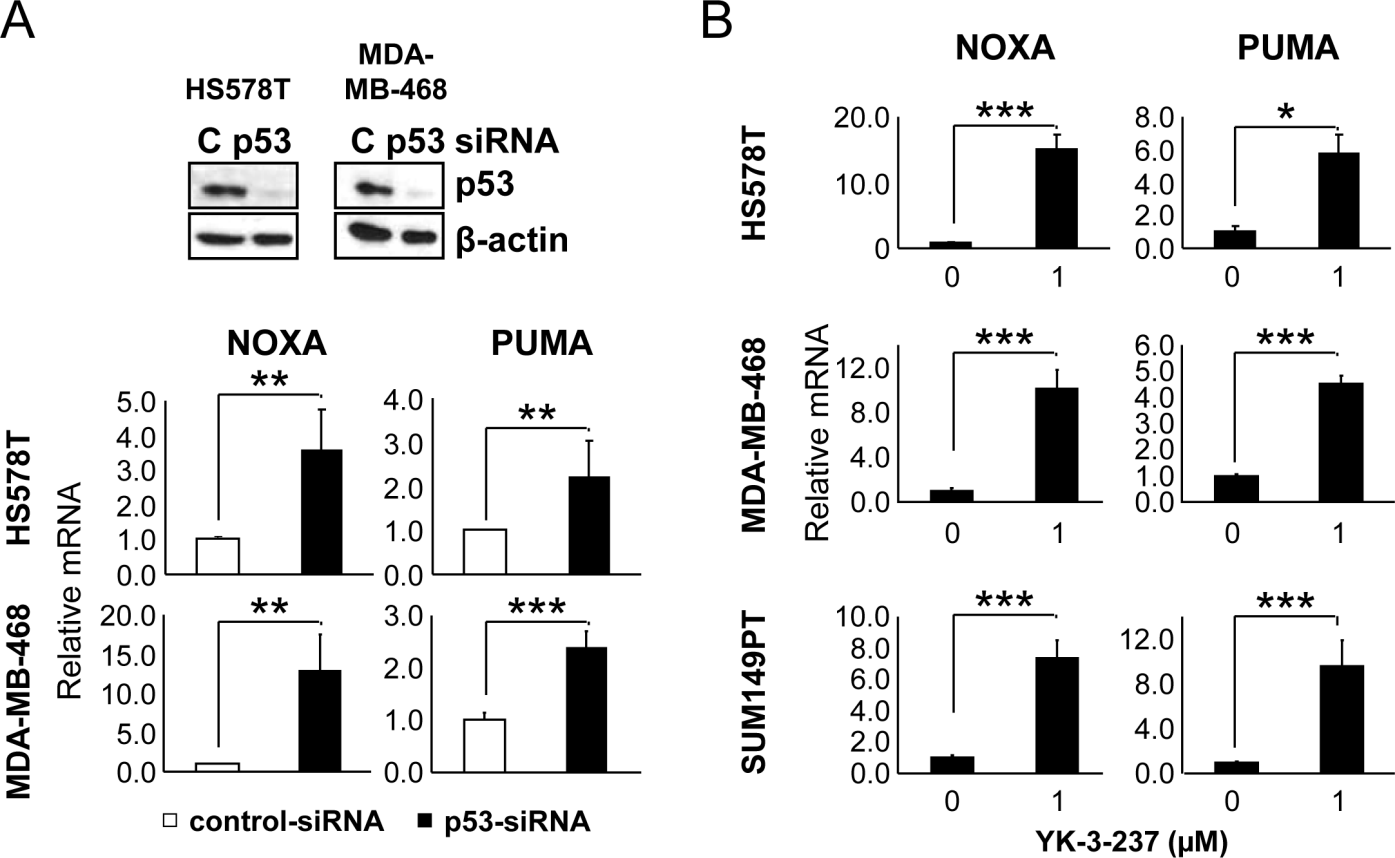
The effect of mtp53 depletion and YK-3-237 on the expression of WTp53-target genes in TNBC cell lines A, the effect of mtp53 knockdown on the expression of WTp53-target genes. Cells were transfected with either control (C)- or p53-siRNA and subjected to quantitative real time-PCR (qRT-PCR) as described in Materials and Methods. Data are shown as mean ±SD. Knockdown of mtp53 was assessed by western blot analysis.**P < 0.01 and ***P < 0.001. B, qRT-PCR analysis of WTp53-target genes in cells treated with YK-3-237. Cells were treated with YK-3-237 for 24 hr and qRT-PCR was performed as in A. Data are presented as mean ±SD. *P < 0.05 and ***P < 0.001.

Previously, it has been reported that mtp53 reduces p63/p73-mediated transcriptional activation of WTp53-target genes such as NOXA, GADD45, p21, BAX, p53AIP-1, IGFBP3, p53R2, cyclin G1, hTERT, and MDM2 [39-42] and knockdown of mtp53 activates expression of WTp53-target genes in a cell type-specific manner [40]. Specifically, knockdown of mtp53 was reported to induce the expression of GADD45, PTEN and PERP mRNA in a hepatoma cell line HUH-7, GADD45 and p21 mRNA in a breast cancer cell line T47D, and p21 mRNA in a nasopharyngeal carcinoma cell line CNE-2 [40]. Consistent with these reports, knockdown of mtp53 in two TNBC cell lines, HS578T and MDA-MB-468, induced the mRNA expression of PUMA and NOXA (Figure 3A). Quantitative real time-PCR analysis further confirmed the YK-3-237-mediated induction of WTp53-target genes, PUMA and NOXA in three different TNBC cell lines, HS578T, MDA-MB-468, and SUM149PT (Figure 3B). These results suggest that the reduction of mtp53 level by YK-3-237-mediated deacetylation functionally and specifically releases transcriptional suppression of WTp53 target genes by mtp53 proteins in these cells.

### YK-3-237 induces apoptotic cell death in TNBC cells

**Figure 4:**
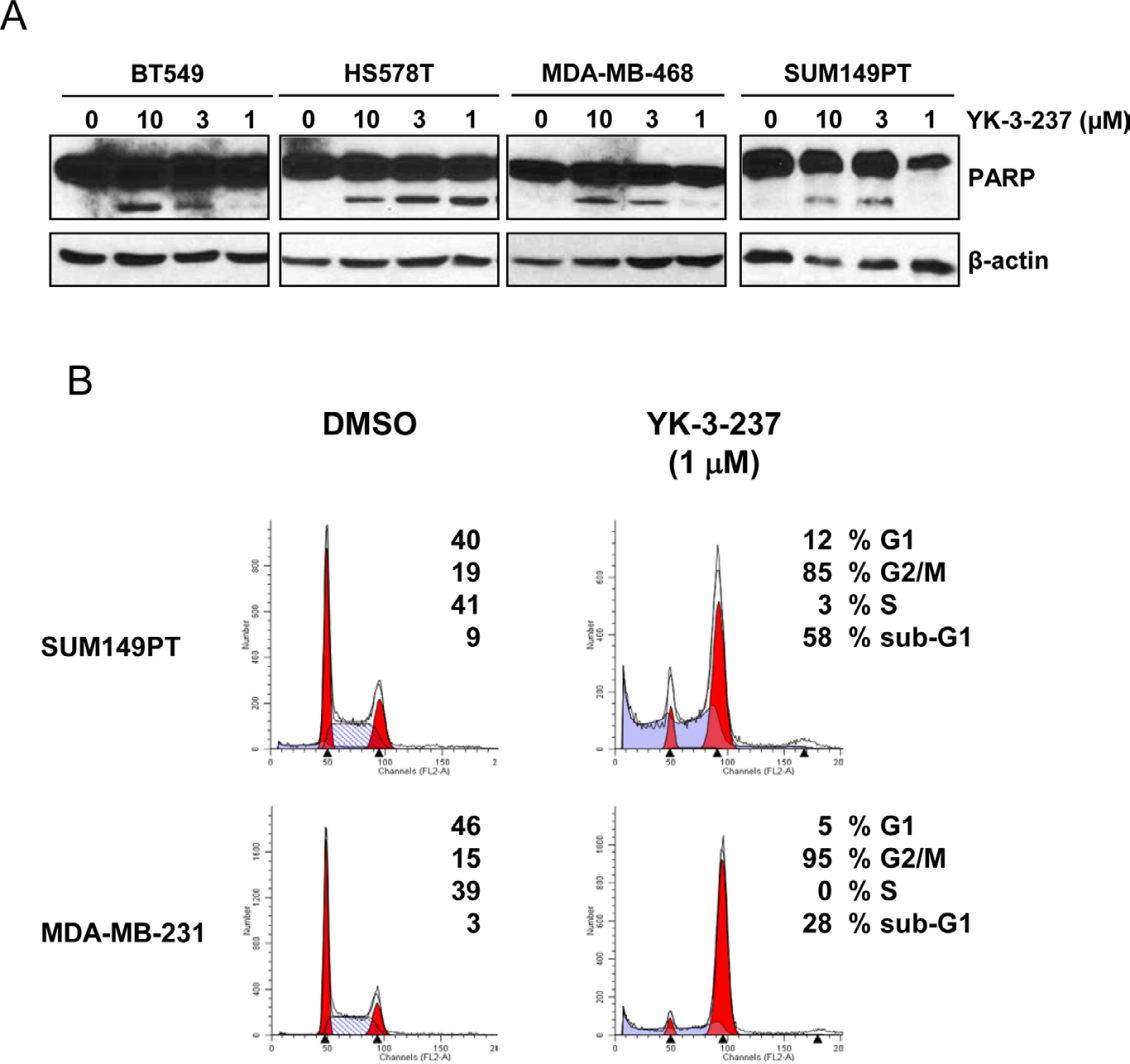
Induction of apoptotic cell death and G2/M arrest by YK-3-237 in TNBC cell lines Cells were treated with YK-3-237 for 24 hr and both floating and attached cells were harvested for western blot analysis (A) and cell cycle analysis (B). In A, β-actin was used as a loading control.

It has been well established that induction of WTp53 target genes arrests cells to G2 phase of cell cycle and finally leads cells to apoptotic cell death [43]. In fact, YK-3-237 induced PARP cleavage, a hallmark of caspase-dependent apoptosis, within 24 hr post-treatment (Figure 4A). TNBC cells treated with YK-3-237 were markedly arrested at G2/M phase (Figure 4B). In addition, sub-G1 fractions were also drastically increased by YK-3-237 within 24 hr post-treatment in TNBC cells with mtp53 (Figure 4B).

## DISCUSSION

In this study we found that the small molecule YK-3-237 reduced the proliferation of a broad range of breast cancer cell lines carrying mtp53. YK-3-237 reduced the level of mtp53 by removing acetyl group from its K382 residue in a SIRT1-dependent manner. In addition, YK-3-237 activated *in vitro* enzyme activity of purified human SIRT1 more potently than resveratrol. In cells carrying WTp53, YK-3-237 enhanced SIRT1-mediated repression of transcriptional activation by WTp53. Similar to mtp53-KD, YK-3-237 induced mRNA expression of PUMA and NOXA and caused apoptotic cell death of TNBC cells.

Since more than 50% of human cancers possess mutations of p53, mtp53 represents a potentially important anti-cancer target [4,6]. More importantly, the active oncogenic potential by gain-of-function of mtp53s stresses this approach as a broad range therapeutic opportunity. Many attempts, however, have failed because of the diverse character of mtp53 [6]. Molecules that restore the wild-type conformation of mtp53 such as PRIMA-1 [44], MIRA-1 [45], CP-31398 [46], and P53R3 [47] have effects on specific types of mtp53. More recently, a HSP90 inhibitor 17AAG has been suggested as an alternative approach to reduce the proliferation of a broad range cancer cell lines carrying various types of mtp53 [48]. In this study, inhibiting HSP90 by 17AAG disrupted the molecular chaperone complex to liberate mtp53 for degradation by MDM2.

The mechanism of hyperstabilization of mtp53 is largely unknown. The stabilization of p53 proteins are thought to be regulated by post-translational modifications. For WTp53, acetylation of K382 residue has been proposed as an important determinant to stabilize the WTp53 protein [33]. Although mtp53 is known to be acetylated [34], the roles of these acetylations of mtp53 still need to further investigation. We demonstrated in this study that mtp53 is acetylated at K382 residue in TNBC cell lines and a small molecule compound YK-3-237 reduced this acetylation of mtp53 and depleted the level of mtp53. In addition, YK-3-237 activated SIRT1 enzyme activity *in vitro* and the deacetylation of either WTp53 or mtp53 was evidently dependent on SIRT1 activity, which was demonstrated by overexpression of wild type or DN-SIRT1, SIRT1-KD, and SIRT1 inhibition by a small molecule inhibitor, regardless of mtp53 types. However, the molecular mechanism of SIRT1 activation by YK-3-237 needs to be further elucidated. As reported recently [29], YK-3-237 could activate *in vitro* SIRT1 activity in the presence of aminomethylcoumarin (AMC)-tagged peptide derived from p53 protein. Contrarily, YK-3-237 could not activate *in vitro* SIRT1 activity toward nascent p53 peptide (data not shown). Allosteric SIRT1 activator such as STAC-1 has been known to require specific hydrophobic motifs found in a subset of SIRT1 substrates including PGC-1α and FOXO3a to facilitate SIRT1 activation [29]. To our knowledge, p53 has no such motifs. Notably, SRT1720 was reported to reduce acetyl-p53 in cells [22], in spite of it could not activate *in vitro* SIRT1 activity toward nascent p53 peptide [28]. How YK-3-237 activates SIRT1 to remove acetyl group from mtp53 in cells needs to be determined in the future.

As we demonstrated, YK-3-237 also activated the *in vitro* enzyme activity of human SIRT2. Additionally, YK-3-237 reduced the acetyl-α-tubulin (K40) and the level of α-tubulin in cells. Because YK-3-237 was determined not to bind tubulin binding sites *in vitro* [31], it is less plausible that YK-3-237 exerts its anti-proliferative effect through inhibition of tubulin polymerization. Rather decrease of α-tubulin through deacetylation by SIRT2 may contribute G2/M arrest in a cooperative manner with depletion of mtp53.

In summary, we demonstrated YK-3-237 is a small molecule activator of SIRT1. Activation of SIRT1 by YK-3-237 functionally reduces the level of mtp53 by deacetylation. The roles of SIRT1 in human cancers are controversial [18,19] and both SIRT1 inhibitors and activators (such as resveratrol) have been reported to inhibit growth of human cancer cell lines [19]. Although several SIRT1 activating compounds have been recently developed [20-23], the anti-cancer effects of these compounds have not been reported. Because SIRT1 has a deacetylase activity against broad range of substrates acting as either tumor promoters or tumor suppressors [18,19], activating its enzyme activity may be beneficial for certain subtypes of cancers. Given increasing interests of SIRT1 in many biological processes including cancer, aging, diabetes, and neuronal diseases, YK-3-237 may represent a valuable tool for basic research and pharmaceutical development.

## MATERIALS AND METHODS

### Cell culture and reagents

The SUM149PT and SUM1315MO2 cell lines were purchased from Asterand (Detroit, MI) and maintained as recommended. All other cell lines were obtained from American Type Cell Culture Collection (Manassas, VA) or from the Tissue Culture Shared Resource of Georgetown University Medical Center. MCF7, MDA-MB-231, and T47D were maintained in Dulbecco's Modified Eagle Medium (DMEM) containing 5% heat inactivated fetal bovine serum (HI-FBS; HyClone, Logan, UT) and 100 units/ml penicillin/streptomycin. HCC1937 and ZR-75-1 cells were maintained in RPMI1640 containing 10% HI-FBS and 100 units/ml penicillin/streptomycin. MDA-MB-468, HS578T, BT549, MDA-MB-453, BT474, and SK-BR-3 cells were maintained DMEM containing 10% HI-FBS and 100 units/ml penicillin/streptomycin. Cell culture reagents were obtained from Lonza (Basel, Switzerland), Invitrogen (Carlsbad, CA), or Cellgro (Manassas, VA). YK-3-237 was synthesized as described previously [31] and dissolved in dimethyl sulfoxide (DMSO).

### MTT (3-(4,5-Dimethylthiazol-2-yl)-2,5-diphenyltetrazolium bromide) assay

Cell viability was measured by MTT assay as described previously [49]. In brief, cells were counted by the Luna Automated Cell Counter (Logos Biosystems, Gyunggi-Do, Korea) and subcultured in 96-well plates. The day after subculture, cells were treated with increasing amount of YK-3-237 and further incubated for ~72 hr in triplicate. To measure the viable cells, 20 µl of 5 mg/ml MTT solution was added per 100 µl of growth media. After 2-4 hr incubation at 37°C, the media were removed and 150 µl/well of DMSO was added to dissolve the formazan. The absorbance was measured by ELx808 microplate reader (BioTek, Winooski, VT). Viable cells are presented as a percent of the control. The half maximal inhibitory concentration (EC_50_) was calculated by CompuSyn software V1.0 (ComboSyn, Paramus, NJ). The relative sensitivity was calculated by equation of – Log2[(EC_50_ of each cell line)/(EC_50_ of T47D)].

### Small interfering RNA (siRNA) transfection

For siRNA transfection, cells were subcultured in 6-well plates with density of 0.6~1 × 10^5^ cells/well. The day after subculture, 100 nM/well of siRNA was transfected by Lipofectamine 2000 reagent (Invitrogen) in serum free media. Four hours after transfection, equal volume of normal growth media was added to each well and the cells were further incubated for 3-days. The siRNAs were purchased from Dharmacon (Lafayette, CO) with following sequences: control-siRNA, 5'-GACGAGCGGCACGUGCACAUU-3'; SIRT1-siRNA, 5'-CCACCUGAGUUGGAUGAUA-3'; p53-siRNA, 5'-GAC UCC AGU GGU AAU CUA CUU-3'.

### In vitro SIRT assay

The Fluor-de-Lys-SIRT1 and SIRT2 deacetylase assay were performed as manufacturer's instruction (Enzo Life Sciences, Farmingdale, NY). For competition assay, 50 µM of suramin was added to reaction.

### p53 reporter gene assay

Plasmid DNAs for reporter gene assay were obtained from following sources: SIRT1 and DN-SIRT1 from Addgene (Cambridge, MA); Expression vector for WTp53 from Dr. B. Vogelstein; and p53-Luc from Promega (Madison, WI). MCF7 cells were transfected with p53-Luc reporter plasmid with expression vector for WTp53 in the presence or absence of SIRT1 expression vector by Lipofectamine 2000 (Invitrogen). Twenty-four hour after transfection, the cells were further treated with YK-3-237 for 24 hr and luciferase activity was measured according to manufacturer's instruction (Promega) using Victor2 plate reader (PerkinElmer, Waltham, MA, USA) at the Genomics and Epigenomics Shared Resource of Georgetown University Medical Center and normalized to β-galactosidase activities.

### Western blot analysis and antibodies

Preparation of cell lysates and western blot analyses were performed as described previously [49]. Antibodies were obtained from the following sources: Ac-p53 (K382; #2525), Ac-α-tubulin (K40; #3971), phospho-ACC (S78; #3661), and ACC (#3676) from Cell Signaling (Danvers, MA); PARP (#556494) from BD Pharmingen (San Jose, CA); p53 (V1003) from Biomeda (Foster City, CA); ERα (sc-543) and SIRT1 (sc-15404) from Santa Cruz (Santa Cruz, CA); α-tubulin, β-actin (A1978), and horseradish peroxidase-conjugated secondary antibodies from Sigma (St. Louis, MO). Chemiluminescence reagent was obtained from Santa Cruz or Thermo Scientific (Rockford, IL).

### Cell cycle analysis

Cells were treated with either DMSO or 1 µM of YK-3-237 for 24 hr. The attached cells were harvested by trypsinization and combined with floating cells in the culture media. After washing by phosphate-buffered saline, the cells were fixed with 70% ethanol at -20˚C. Flow cytometric analysis was performed with a FACSCalibur flow cytometer (Becton-Dickinson, CA) at the Flow Cytometry and Cell Sorting Shared Resource at Georgetown University Medical Center.

### Quantitative real time-PCR (qRT-PCR) analysis

qRT-PCR was performed as described previously [50] with an Applied Biosystems-Prism Sequence Detector System 7700 at the Genomics and Epigenomics Shared Resource of Georgetown University Medical Center. The primers were used with following sequences: PUMA: forward, 5´-GAC CTC AAC GCA CAG TAC-3´ and reverse, 5´-GCA TCT CCG TCA GTG CAC-3´; NOXA: forward, 5´-TCC GGC AGA AAC TTC TGA AT-3 and reverse, 5´-TTC CAT CTT CCG TTT CCA AG-3´; GAPDH: forward, 5'-GTA TGA CAA CGA ATT TGG CTA CAG-3' and reverse, 5'-AGC ACA GGG TAC TTT ATT GAT GGT-3'.

### Statistical analysis

To compare two groups of interest, the two-tailed Student's t-test was applied for statistical analysis. * indicates P < 0.05; ** indicates P < 0.01; and *** indicates P < 0.001.

## SUPPLEMENTARY FIGURES


