# Supplementary material for: Targeting Mutant p53 by a SIRT1 Activator YK-3-237 Inhibits the Proliferation of Triple-Negative Breast Cancer Cells
Source: Oncotarget. 2013 Jul 5;4(7):984–94. doi: 10.18632/oncotarget.1070 (PMC3759676; doi:10.18632/oncotarget.1070)
Supplement: Supplementary file 1 [file oncotarget-04-984-s001.ppt]

## Slide 1
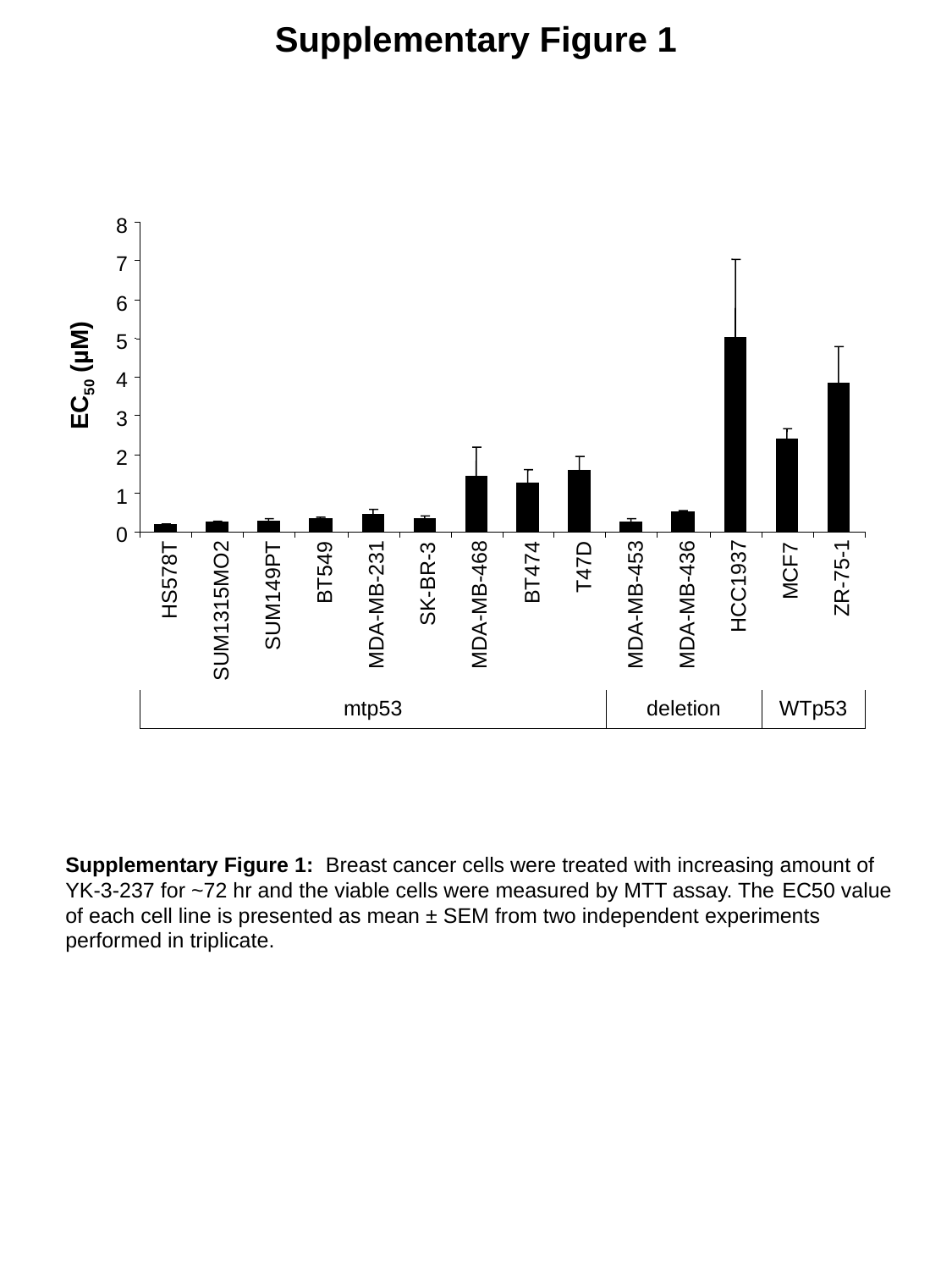

Supplementary Figure 1
8
7
6
5
EC50 (µM)
4
3
2
1
0
T47D
MCF7
BT549
BT474
ZR-75-1
HS578T
SK-BR-3
HCC1937
SUM149PT
MDA-MB-231
MDA-MB-468
MDA-MB-453
MDA-MB-436
SUM1315MO2
| mtp53 | deletion | WTp53 |
| --- | --- | --- |
| | | |
Supplementary Figure 1: Breast cancer cells were treated with increasing amount of YK-3-237 for ~72 hr and the viable cells were measured by MTT assay. The EC50 value of each cell line is presented as mean ± SEM from two independent experiments performed in triplicate.

## Slide 2
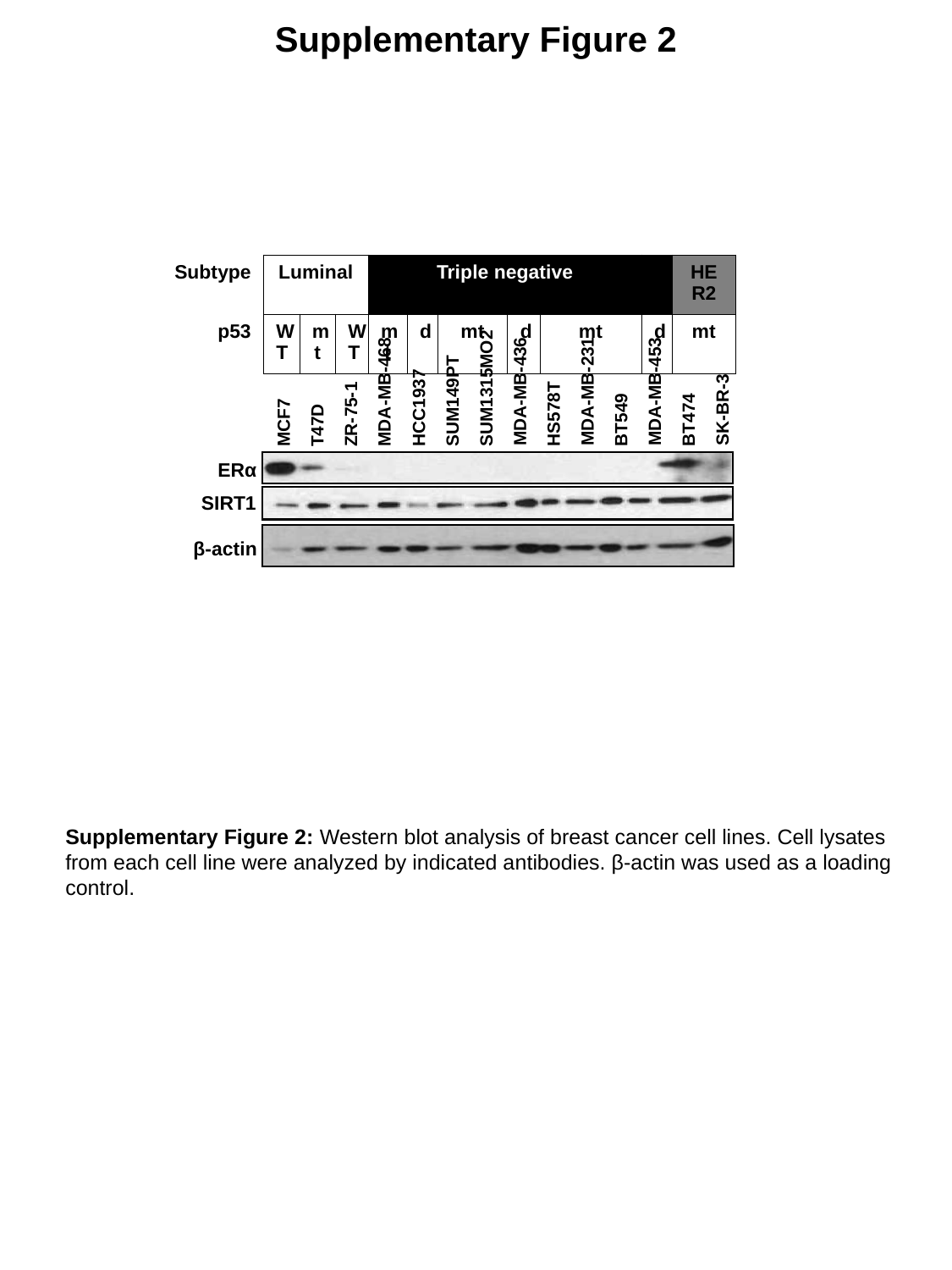

Supplementary Figure 2
| Subtype | Luminal | | | Triple negative | | | | | | HER2 |
| --- | --- | --- | --- | --- | --- | --- | --- | --- | --- | --- |
| p53 | WT | mt | WT | mt | d | mt | d | mt | d | mt |
SUM1315MO2
MDA-MB-231
MDA-MB-453
MDA-MB-436
MDA-MB-468
SUM149PT
HCC1937
SK-BR-3
HS578T
ZR-75-1
BT549
BT474
MCF7
T47D
ERα
SIRT1
 β-actin
Supplementary Figure 2: Western blot analysis of breast cancer cell lines. Cell lysates from each cell line were analyzed by indicated antibodies. β-actin was used as a loading control.

## Slide 3
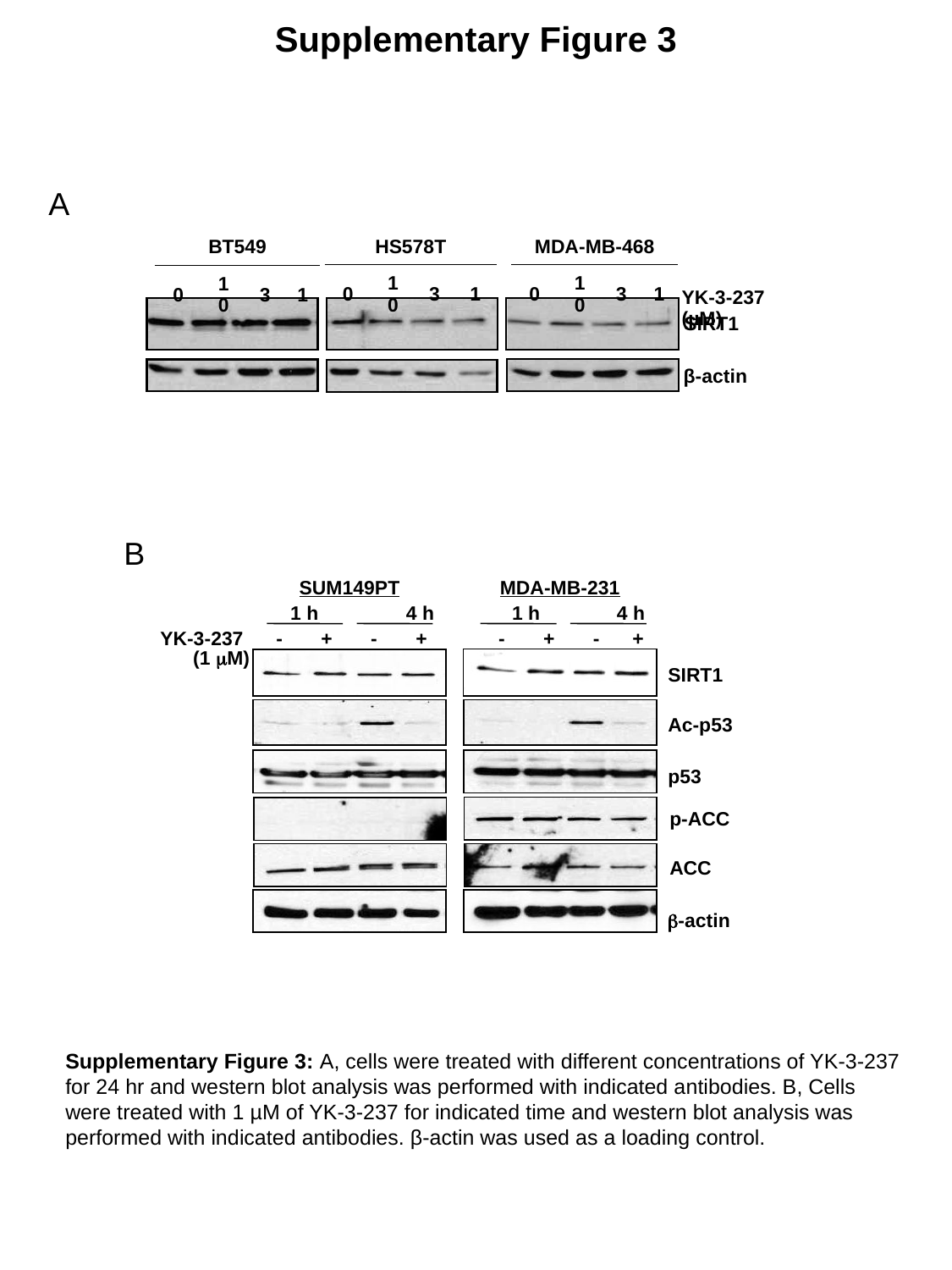

Supplementary Figure 3
A
| HS578T | | | |
| --- | --- | --- | --- |
| 0 | 10 | 3 | 1 |
| MDA-MB-468 | | | |
| --- | --- | --- | --- |
| 0 | 10 | 3 | 1 |
| BT549 | | | |
| --- | --- | --- | --- |
| 0 | 10 | 3 | 1 |
| |
| --- |
| YK-3-237 (µM) |
 SIRT1
 β-actin
B
SUM149PT
MDA-MB-231
1 h 4 h
1 h 4 h
YK-3-237 - + - + - + - +
(1 μM)
SIRT1
Ac-p53
p53
p-ACC
ACC
β-actin
Supplementary Figure 3: A, cells were treated with different concentrations of YK-3-237 for 24 hr and western blot analysis was performed with indicated antibodies. B, Cells were treated with 1 µM of YK-3-237 for indicated time and western blot analysis was performed with indicated antibodies. β-actin was used as a loading control.

## Slide 4
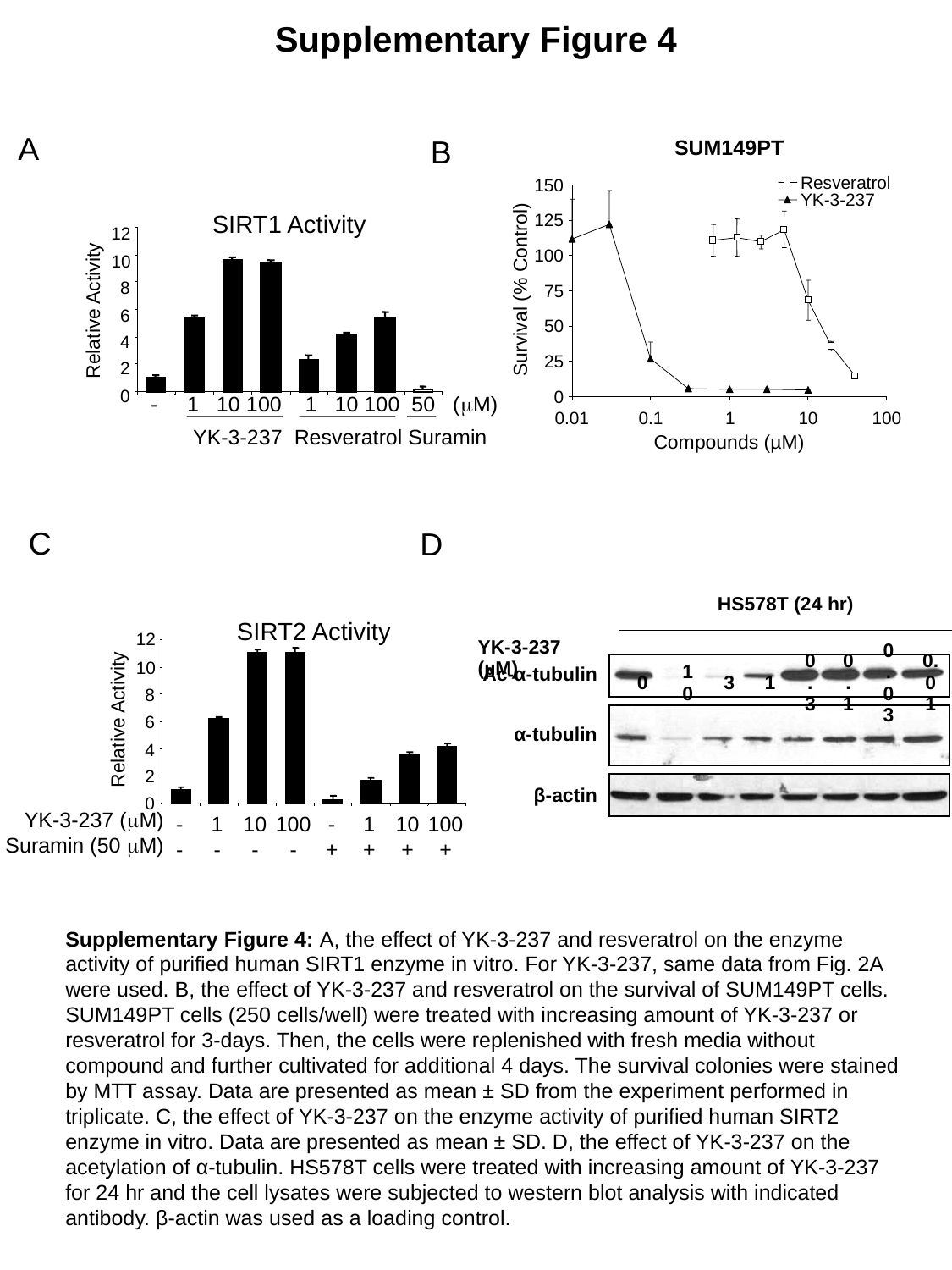

Supplementary Figure 4
A
B
SUM149PT
Resveratrol
150
YK-3-237
SIRT1 Activity
125
12
10
100
8
Survival (% Control)
75
Relative Activity
6
50
4
25
2
0
- 1 10 100 1 10 100 50 (μM)
0
0.01
0.1
1
10
100
YK-3-237 Resveratrol Suramin
Compounds (µM)
C
D
| | HS578T (24 hr) | | | | | | | |
| --- | --- | --- | --- | --- | --- | --- | --- | --- |
| YK-3-237 (µM) | 0 | 10 | 3 | 1 | 0.3 | 0.1 | 0.03 | 0.01 |
SIRT2 Activity
12
10
 Ac-α-tubulin
8
Relative Activity
6
 α-tubulin
4
2
 β-actin
0
YK-3-237 (μM)
Suramin (50 μM)
-
-
1
-
10
-
100
-
-
+
1
+
10
+
100
+
Supplementary Figure 4: A, the effect of YK-3-237 and resveratrol on the enzyme activity of purified human SIRT1 enzyme in vitro. For YK-3-237, same data from Fig. 2A were used. B, the effect of YK-3-237 and resveratrol on the survival of SUM149PT cells. SUM149PT cells (250 cells/well) were treated with increasing amount of YK-3-237 or resveratrol for 3-days. Then, the cells were replenished with fresh media without compound and further cultivated for additional 4 days. The survival colonies were stained by MTT assay. Data are presented as mean ± SD from the experiment performed in triplicate. C, the effect of YK-3-237 on the enzyme activity of purified human SIRT2 enzyme in vitro. Data are presented as mean ± SD. D, the effect of YK-3-237 on the acetylation of α-tubulin. HS578T cells were treated with increasing amount of YK-3-237 for 24 hr and the cell lysates were subjected to western blot analysis with indicated antibody. β-actin was used as a loading control.
